# Syndrome de Rapunzel chez une enfant trisomique 21: à propos d’un cas

**DOI:** 10.11604/pamj.2022.42.230.36268

**Published:** 2022-07-26

**Authors:** Rabab Atae-Allah, Yousra El Boussaadni, Kawtar Khabbache, Saad Andaloussi, Aziz Elmadi, Abdallah Oulmaati

**Affiliations:** 1 Service de Pédiatrie, Centre Hospitalier Universitaire Tanger, Université Abdelmalek Essaadi, Tétouan, Maroc,; 2 Service de Chirurgie Pédiatrique, Centre Hospitalier Universitaire, Université Abdelmalek Essaadi, Tétouan, Maroc

**Keywords:** Bézoard, syndrome de Rapunzel, trisomie, cas clinique, Bezoar, Rapunzel syndrome, trisomy, case report

## Abstract

Le trichobezoard est une pathologie rare, elle correspond à la présence de cheveux et ou de fibres au niveau du tube digestif, conséquence d´une attitude compulsive (trichotillomanie) et d´un trouble de comportement alimentaire (trichophagie). Le trichobézoard gastrique est le plus fréquent, peut s´étendre à l´intestin grêle arrivant parfois à la dernière anse iléale, voire au côlon transverse, réalisant ainsi le syndrome de Rapunzel. Nous rapportons un cas de trichobézoard gastroduodénal et grêlique chez une jeune fille de 6 ans avec faciès trisomique, consulte pour des douleurs abdominales récidivantes depuis 01 mois avec une suspicion de lymphome digestif chez qui le diagnostic de trichobézoard est fait en per opératoire. Notre but est de donner un aperçu sur l´historique de cette affection rare et de préciser les modalités diagnostiques et thérapeutiques.

## Abstract (en)

Trichobezoard is a rare condition characterized by a gastric mass composed of hair or fibers due to a compulsive attitude (trichotillomania) and an eating disorder (trichophagia). Gastric trichobezoar is the most common form and may extend into the small bowel, sometimes reaching the last ileal loop, or even the transverse colon, resulting in Rapunzel syndrome. We here report a case of gastroduodenal and small intestine trichoboozoar in a 6-year-old girl with facies of trisomy, presenting with recurrent abdominal pain lasting for one months and suspected gastrointestinal lymphoma. The diagnosis of trichoboozoar was based on surgery. The purpose of this study is to give an overview of the history of this rare condition and to clarify the diagnostic and therapeutic approaches used.

## Introduction

Le syndrome de Rapunzel a été décrit pour la première fois par Vaughan *et al*. en 1968, il correspond à une forme exceptionnelle de bézoards. La définition de cette pathologie n´est pas univoque. Elle est décrite comme l´extension à travers le pylore d´un bézoard gastrique qui peut arriver jusqu´au cæcum ou comme l´association d´un bézoard gastrique et grêlique ou finalement comme un bézoard intestinal associé à une occlusion digestive plus ou moins complète [1]. En rapportant cette observation récente caractéristique de cette forme anatomique, nous mettons le point sur la particularité du contexte de notre patiente trisomique, les difficultés diagnostiques rencontrées, les modes de révélation et surtout la gravité potentielle de cette affection.

## Patient et observation

**Informations de la patiente**: il s´agit d´une fille âgée de 6 ans, avec un faciès trisomique, sans antécédents pathologiques notables qui présente des douleurs abdominales intermittentes associées à des vomissements alimentaires précoces évoluant depuis un mois, l´enfant est mise sous traitement symptomatique sans amélioration.

**Résultats cliniques**: l´enfant est apyrétique stable sur le plan hémodynamique et respiratoire, avec une distension et une sensibilité abdominales diffuses sans trouble de transit.

**Figure 1:**
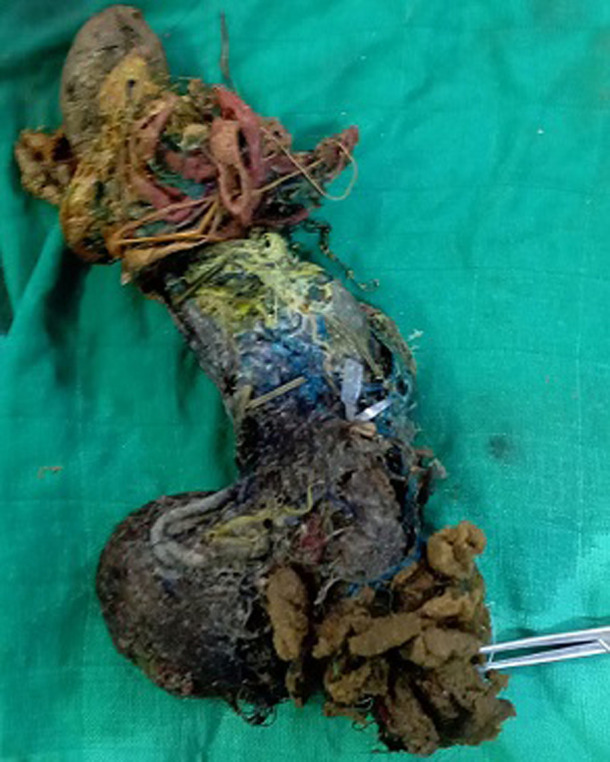
trichobézoard et ses prolongements duodénaux et jéjunaux

**Démarche diagnostique**: l´échographie abdominale évoque un aspect en faveur d´un lymphome digestif devant l´épaississement digestif compliqué d´une invagination secondaire. La décision était de compléter l´exploration par la réalisation d´un scanner abdominal avec injection de produit de contraste, qui a objectivé un bezoard gastroduodénal et grêlique avec épaississement pariétal digestif et invagination grêlo-grêlique au niveau du flanc droit (Figure 1). Le bilan biologique objective un taux d´hémoglobine normal à 12,6 g/dl, une protéine C-réactive (CRP) élevée à 169,7 mg/l, une hyperleucocytose à 12 340/mm^3^ à prédominance à polynucléaires neutrophiles (PNN): 8820/mm^3^; une vitesse de sédimentation accélérée à 59 à la première heure, la ferritinémie et la vitamine B12 sont correctes.

**Figure 2:**
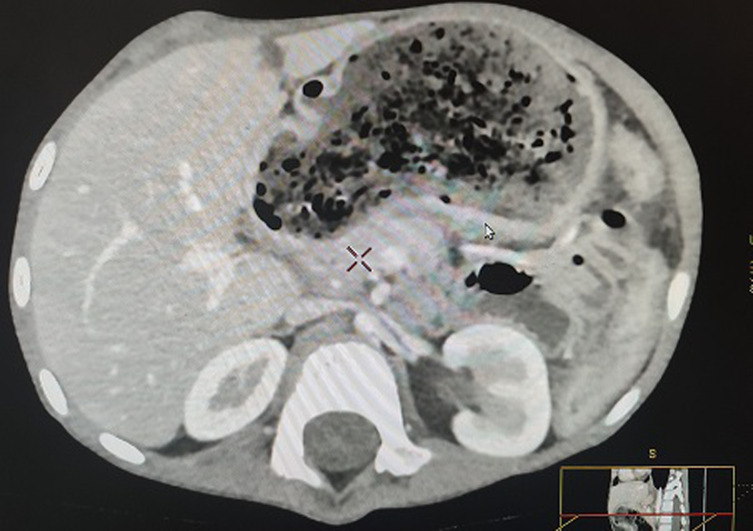
scanner abdominal avec injection de produit de contraste montre un contenu des anses formé de bulles d´air confinées dans une matière dense identique à celle de l´estomac en rapport avec le bézoard

**Intervention thérapeutique et suivi**: une laparotomie médiane a été réalisée en urgence confirmant le diagnostic. La gastrotomie a permis l´extraction d´un trichobézoard d´environ 20 cm de largeur (Figure 2) et de ses prolongements duodénaux et jéjunaux. Il était marron verdâtre, contenant des cheveux et des fibres d´origine variable. Ces constatations sont typiques d´un syndrome de Rapunzel défini par l´existence d´un trichobézoard gastrique avec extension duodénale et jéjunale. Les suites opératoires sont simples, en reprenant l´interrogatoire avec la famille, la maman a rapporté la notion de trouble de comportement alimentaire type trichophagie associé à une auto-agressivité évoluant depuis un an.

**Consentement éclairé**: le consentement a été obtenu auprès de la famille du patient.

## Discussion

Le trichobézoard est une affection rare chez l´enfant (0,15% des corps étrangers gastrointestinaux), le plus souvent diagnostiquée à un âge tardif avec un pic entre 10 et 19 ans [2]; cette atteinte est plus fréquente chez le sexe féminin (90% des cas), et elle touche de grands enfants dans la majorité des cas [3]. Le premier cas de bézoard a été décrit en 1779, et jusqu´à nos jours, il s´observe en particulier chez les malades présentant des troubles psychiatriques notamment le syndrome de Pica ou chez les patients ayant subi une gastrectomie partielle [4]. Notre observation peut s´intégrer chez une patiente avec des troubles de conduite alimentaire dans le cadre de sa trisomie. La localisation gastrique du trichobézoard est la plus fréquente, les boucles de cheveux ingérées sont attrapées par la muqueuse à laquelle elles se fixent et forment une sorte de grillage au niveau duquel s´agglomèrent les aliments, réalisant une masse compacte fixée à la paroi gastrique. Le trichobézoard formé peut s´étendre à l´intestin grêle arrivant parfois à la dernière anse iléale [5], voire au côlon transverse, réalisant ainsi le syndrome de Rapunzel [6], comme le cas chez notre patiente qui présente un trichobézoard étendu de l´estomac à l´intestin grêle.

Cette atteinte peut rester longtemps asymptomatique, ce qui explique le retard du diagnostic qui va dans certains cas jusqu´à plusieurs années. La symptomatologie clinique est très variable, et non spécifique [7]. Les signes digestifs sont au premier plan et comportent des douleurs abdominales diffuses ou plus spécifiquement épigastriques, nausées, vomissements, troubles de transit type diarrhée ou constipation, œsophagite peptique et parfois une mauvaise haleine par putréfaction alimentaire, l´anorexie et l´amaigrissement sont parfois l´élément clinique unique. Dans certains cas, elle peut être révélé d´emblée par une complication comme une hémorragie digestive, une occlusion intestinale aiguë, une perforation digestive, un ictère cholestatique, un ulcère gastrique ou duodénal et rarement un volvulus du gros intestin, ou une pancréatite aiguë imputée à une obstruction de l´ampoule de Vater par un prolongement du trichobézoard [6-9]. A l´examen clinique, en dehors des complications, on peut trouver une masse abdominale localisée le plus souvent au niveau de l´hypocondre gauche et/ou de l´épigastre [3], il s´agit d´un signe spécifique inconstant qui peut manquer dans certaines situations comme dans le cas de notre malade.

La présence d´une plaque d´alopécie localisée est un signe d´orientation important qui doit nous pousser à rechercher une trichotillomanie [7], chez notre patiente l´examen du cuir chevelu était sans particularité. Sur le plan biologique, on peut trouver une anémie hypochrome microcytaire, une hyperleucocytose, une hypoalbuminémie, une férritinémie et un taux de vitamine B12 bas [8]. Lorsque le diagnostic est évoqué, l´examen complémentaire de choix est la fibroscopie œsogastro-duodénale, qui a un intérêt à la fois diagnostique et thérapeutique dans les formes localisées gastriques et de petite taille, elle permet donc de confirmer le diagnostic et d´extraire le corps étranger. Elle permet de visualiser des cheveux enchevêtrés de couleur noirâtre mais généralement une modification de couleur a lieu due à l´effet chimique de l´acidité gastrique cette constatation est pathognomonique du trichobézoard [8,10].

Lorsque le trichobézoard s´étend à l´intestin grêle arrivant parfois à la dernière anse iléale, voire au côlon transverse on parle de syndrome de Rapunzel. La tomodensitométrie abdominale montre une masse dans la lumière digestive formée de bulles d´air confinées dans une matière dense hétérogène qui moule la paroi. Le scanner montre généralement un épaississement et une prise de contraste inflammatoire de la paroi digestive [11]. Le traitement nécessite une prise en charge chirurgicale initiale et un support psychiatrique nécessaire. L´extraction est souvent chirurgicale par entérotomie ou gastrotomie selon la localisation du trichobézoard. Il est nécessaire d´explorer tout le tube digestif à la recherche de localisations multiples. En cas de nécrose intestinale, il faut réaliser de multiples entérotomies pour réduire les risques de perforation au moment de l´extraction du bézoard. L´extraction endoscopique ou par lavage gastrique des petits bézoards est possible mais dans le cas syndrome de Rapunzel le traitement est toujours chirurgical comme c´était le cas chez notre patiente ayant nécessité une intervention chirurgicale [12]. La trichophagie a été rapportée dans le cadre de troubles psychotiques ou autistiques [13]. D´où la nécessité d´une prise en charge psychiatrique, à base de thérapie comportementale, d´éducation parentale et de traitement médical [14]. Les thérapies cognitivo-comportementales ont approuvé leur efficacité alors que le traitement médicamenteux (type inhibiteur sélectif de la recapture de la sérotonine) s´avère peu efficace [15].

## Conclusion

Le trichobézoard est une pathologie rare qui survient habituellement chez des enfants présentant des troubles psychiques ou sur terrain particulier comme c´est le cas de notre malade porteuse d´une trisomie 21. Les signes cliniques sont digestifs souvent associés à des signes généraux. Le diagnostic fait appel à la radiologie notamment l´échographie, Le scanner, le transit œso-gastro-duodénal (TOGD) et l´endoscopie. En plus de la chirurgie, la prise en charge psychologique est un temps essentiel dans le traitement et surtout dans la prévention des récidives. Le syndrôme de Rapunzel a été largement décrit en littérature en précisant ses modalités diagnostiques et thérapeutiques, cependant sa rareté chez l´enfant et le contexte particulier de notre malade trisomique représentent l´intérêt de notre observation.
